# Supplementary material for: Analysis of the immune response to sciatic nerve injury identifies efferocytosis as a key mechanism of nerve debridement
Source: eLife. 2020 Dec 2;9:e60223. doi: 10.7554/eLife.60223 (PMC7735761; doi:10.7554/eLife.60223)
Supplement: Transparent reporting form [file elife-60223-transrepform.docx]

***eLife’s* transparent reporting form**

We encourage authors to provide detailed information *within their submission* to facilitate the interpretation and replication of experiments. Authors can upload supporting documentation to indicate the use of appropriate reporting guidelines for health-related research (see [EQUATOR Network](http://www.equator-network.org/%20)), life science research (see the [BioSharing Information Resource](https://biosharing.org/)), or the [ARRIVE guidelines](http://www.plosbiology.org/article/info:doi/10.1371/journal.pbio.1000412) for reporting work involving animal research. Where applicable, authors should refer to any relevant reporting standards documents in this form.

If you have any questions, please consult our Journal Policies and/or contact us: [editorial@elifesciences.org](mailto:editorial@elifesciences.org).

**Sample-size estimation**

- You should state whether an appropriate sample size was computed when the study was being designed
- You should state the statistical method of sample size computation and any required assumptions
- If no explicit power analysis was used, you should describe how you decided what sample (replicate) size (number) to use

Please outline where this information can be found within the submission (e.g., sections or figure legends), or explain why this information doesn’t apply to your submission:

Sample size for experiments, biological and technical replicates, were based on previously published work by the authors and historical data. Details on statistical methods for flow cytometry (Figure 1, 2, 3, 6, 7 and 8) are provided in figure legends and material and method sections, as appropriate. A P value of < 0.05 was deemed to be statistically significant. Details on statistical methods for bulk RNAseq and scRNAseq analysis (Figure 4, 5, 6, 7, 8) are provided in material and methods. Statistical methods for axon regeneration in vivo and neurite outgrowth in vitro (Figure 8) are described in the figure legend.

**Replicates**

- You should report how often each experiment was performed
- You should include a definition of biological versus technical replication
- The data obtained should be provided and sufficient information should be provided to indicate the number of independent biological and/or technical replicates
- If you encountered any outliers, you should describe how these were handled
- Criteria for exclusion/inclusion of data should be clearly stated
- High-throughput sequence data should be uploaded before submission, with a private link for reviewers provided (these are available from both GEO and ArrayExpress)

Please outline where this information can be found within the submission (e.g., sections or figure legends), or explain why this information doesn’t apply to your submission:

Throughout the manuscript, the number of biological replicates (n) is provided. For example, for flow cytometry nerves and DRGs from 2-4 animals were pools for one biological replicate. The definition of biological replicate is clearly spelled out in material and methods. The number of biological replicates can be found in the figure legends.

All data were included even outliers for all figures; no data were thrown out, so outliers or exclusion criteria are not explicitly discussed in text.

DRG bulk RNAseq datasets and sciatic nerve scRNAseq datasets have been deposited in GEO database (**GSE153762**).

**Statistical reporting**

- Statistical analysis methods should be described and justified
- Raw data should be presented in figures whenever informative to do so (typically when N per group is less than 10)
- For each experiment, you should identify the statistical tests used, exact values of N, definitions of center, methods of multiple test correction, and dispersion and precision measures (e.g., mean, median, SD, SEM, confidence intervals; and, for the major substantive results, a measure of effect size (e.g., Pearson's r, Cohen's d)
- Report exact p-values wherever possible alongside the summary statistics and 95% confidence intervals. These should be reported for all key questions and not only when the p-value is less than 0.05.

Please outline where this information can be found within the submission (e.g., sections or figure legends), or explain why this information doesn’t apply to your submission:

Statistical analysis methods are described in materials and methods and in figure legends. Raw data are presented in figures; data are shown +/- SEM, sample size and statistical methods are clearly spelled out. P-values are reported.

(For large datasets, or papers with a very large number of statistical tests, you may upload a single table file with tests, Ns, etc., with reference to sections in the manuscript.)

**Group allocation**

- Indicate how samples were allocated into experimental groups (in the case of clinical studies, please specify allocation to treatment method); if randomization was used, please also state if restricted randomization was applied
- Indicate if masking was used during group allocation, data collection and/or data analysis

Please outline where this information can be found within the submission (e.g., sections or figure legends), or explain why this information doesn’t apply to your submission:

Group allocation is discussed throughout the text and in each figure legend. When analysis was blinded, e.g. for axon regeneration in the spinal cord, it is discussed in the materials and methods section.

**Additional data files (“source data”)**

- We encourage you to upload relevant additional data files, such as numerical data that are represented as a graph in a figure, or as a summary table
- Where provided, these should be in the most useful format, and they can be uploaded as “Source data” files linked to a main figure or table
- Include model definition files including the full list of parameters used
- Include code used for data analysis (e.g., R, MatLab)
- Avoid stating that data files are “available upon request”

Please indicate the figures or tables for which source data files have been provided:

Source files are provided for data shown in Figures 4, 5-9.

In addition, we included Figure 5 – source data 1, a file listing the top 100 cell cluster enriched genes identified by scRNAseq (with p-values).
